# Dual Branch Attention Network for Person Re-Identification

**DOI:** 10.3390/s21175839

**Published:** 2021-08-30

**Authors:** Denghua Fan, Liejun Wang, Shuli Cheng, Yongming Li

**Affiliations:** College of Information Science and Engineering, Xinjiang University, Urumqi 830046, China; 1225179654@stu.xju.edu.cn (D.F.); slcaydxju@stu.xju.edu.cn (S.C.); lym@xju.edu.cn (Y.L.)

**Keywords:** person re-identification, deep learning, attention, dual branch, loss function

## Abstract

As a sub-direction of image retrieval, person re-identification (Re-ID) is usually used to solve the security problem of cross camera tracking and monitoring. A growing number of shopping centers have recently attempted to apply Re-ID technology. One of the development trends of related algorithms is using an attention mechanism to capture global and local features. We notice that these algorithms have apparent limitations. They only focus on the most salient features without considering certain detailed features. People’s clothes, bags and even shoes are of great help to distinguish pedestrians. We notice that global features usually cover these important local features. Therefore, we propose a dual branch network based on a multi-scale attention mechanism. This network can capture apparent global features and inconspicuous local features of pedestrian images. Specifically, we design a dual branch attention network (DBA-Net) for better performance. These two branches can optimize the extracted features of different depths at the same time. We also design an effective block (called channel, position and spatial-wise attention (CPSA)), which can capture key fine-grained information, such as bags and shoes. Furthermore, based on ID loss, we use complementary triplet loss and adaptive weighted rank list loss (WRLL) on each branch during the training process. DBA-Net can not only learn semantic context information of the channel, position, and spatial dimensions but can integrate detailed semantic information by learning the dependency relationships between features. Extensive experiments on three widely used open-source datasets proved that DBA-Net clearly yielded overall state-of-the-art performance. Particularly on the CUHK03 dataset, the mean average precision (mAP) of DBA-Net achieved 83.2%.

## 1. Introduction

The Re-ID task aims to search for the most likely images belonging to the same pedestrian in the gallery (candidate image sets). Common challenges include background interference, angle of view change, light intensity, body posture change and occlusion [1].

Recently, algorithms based on deep learning [2,3,4,5,6,7,8] in the Re-ID direction have made significant progress. One of the development trends of related algorithms is using attention mechanism to capture global and local features. Specifically, global features can directly represent the changes of appearance and spatial position of an image. Yang et al. [4] discovered that combining the features of local and global can capture more semantic information. However, global features also consist of the interference information (the background, etc.) due to the lack of local features. 

To solve this problem, Chen et al. [5] proposed the mixed high-order attention network (MHN) to model and utilize the complex high-order statistical information in the attention mechanism. Chen et al. [6] proposed attention but diverse network (ABD-Net) to combine attention modules as a complementary mechanism. 

**Figure 1:**
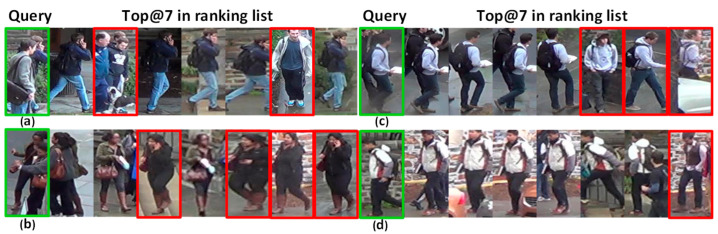
Top@7 in the ranking list of BagTricks [9] on DukeMTMC-ReID. The green boxes are the target pedestrian images, while the red boxes are the wrong retrieval.

The problems of occlusion, similar shape and size changes make retrieval extremely challenging. For Figure 1a, most of the labeled pedestrian’s body is covered. For Figure 1b,c, the labels to be retrieved are similar to nearby people to a great extent. The target pedestrian in Figure 1d is interfered with by background information. Luo et al. [9] proposed a strong baseline with six tricks (including warmup learning rate, random erasing augmentation, label smoothing, last stride, batch normalization neck (BNNeck), and center loss). Woo et al. [10] extended the receiving by a larger convolution kernel. 

Luo et al. [11] proposed that the model’s practical field is only a tiny part of the theoretical acceptance domain. We designed the DBA-Net to capture apparent features of various dimensions. We are committed to integrating attention modules and normalizing the network with a dual branch strategy. 

In particular, our CPSA block integrates global semantic information into local features and focuses on local information between feature maps. Specifically, this module includes a channel attention module (CAB), position attention module (PAB) and spatial attention module (SAB). We observed that CAB can focus on the feature differences of different channels, PAB can learn the dependency of different feature maps through dimensional transformation, and finally SAB integrates apparent global features and plain details of channel, position, and spatial dimensions.

Loss functions play an essential role in optimizing and updating model parameters in backpropagation. Generally, researchers [1,6,10,12] use the loss function of cross-entropy (ID loss) and metric losses to optimize their models. ID loss can correctly assign pedestrians to their classes. Meanwhile, the primary characteristic of Re-ID task is of retrieving the most similar image to the target pedestrian from the gallery. We introduce metric learning, which can autonomously learn the metric distance for a specific task. By calculating the similarity between two images, they can classify the input image into the image category with high similarity. 

Some researchers [6,13,14] chose to use the triplet loss function and have achieved good results. In this paper, we also use triplet loss to enhance the ranking performance. Wang et al. [15] posed a rank list loss (RLL). Since there are hard-negative samples, some negative samples are very similar to the input image, and the network is likely to classify them into the range of positive samples. Gong et al. [16] designed WRLL to solve this problem. In this article, we use ID loss, triplet loss and WRLL to optimize DBA-Net.

In summary, our work has the following contributions:

- We design a residual attention block (CPSA) to extract channel, position and spatial features in different dimensions. It can capture key fine-grained information, such as bags and shoes. We observe that these three blocks are complementary and can capture the vital information of the input image adaptively.
- We notice that the network with multi-branches was more effective in learning robust features than single ones. Specifically, we not only use the global network branch but also introduce another branch after the fourth layer of ResNet-50. Each branch utilizes generalized mean pooling (GeM) [17]. The double branch strategy is significant for fusing global and local vital information.
- Based on ID loss, we use complementary triplet loss and WRL loss to optimize DBA-Net. Each branch employs the same loss function. Triplet loss and WRL loss can enhance the intra-class compactness and inter-class separability in the Euclidean space.

A large number of experiments on Market1501 [18], DukeMTMC-ReID [19] and CUHK03 [2] proved DBA-Net is superior to the state-of-the-art network. Especially on the CUHK03 dataset, the mAP of DBA-Net achieved 83.2%.

The paper is organized as follows: In Section 2, the relevant background to our research is introduced. In Section 3, we present our algorithm in detail. In Section 4, we show the comparative and ablation experiments on these three popular open-source datasets. In Section 5, we visualize and analyze the proposed model. Our overall conclusions are then presented in Section 6.

## 2. Related Work

In this section, we present some outstanding methods related to our network and give a brief introduction to them.

### 2.1. Attention Mechanisms in Person Re-ID 

A host of classic methods can train effective discriminant models [4,20,21,22,23]. Pyramid [24], PCB [25] and MGN [20] achieved advanced performance by integrating global characteristics and local features based on different stripes. Zheng et al. [24] noticed that both global information and local information of the image are vital and need to be used together. Sun et al. [25] combined soft-segmentation (the attention of a neural network) with hard-segmentation (preprocessing and blocking directly), which as better than algorithms only using soft-segmentation or hard-segmentation. Wang et al. [20] directly divided the input image into several horizontal strips, emphasizing that we obtain semantic information not only from meaningful regions. 

Simultaneously, there are problems regarding information redundancy and over-fitting due to the complexity of models. Global features are responsible for the extraction of overall features, and each block of image segmentation is responsible for the extraction of different levels of features. Therefore, there is redundant information between global features and local features.

To diminish the redundancy of a Re-ID network, some researchers [5,8,9,23,26,27,28] focused on models with attention blocks. Tai et al. [9] proposed an attribute attention network (AANet), which integrates information of pedestrians’ critical parts in the network. Zhang et al. [27] presented second-order nonlocal attention (SONA), which can connect local information through second-order features. Li et al. [8] posed harmonious attention (HA), which introduced a cross attention interactive learning mechanism to further optimize attention selection and feature learning. Lin et al. [28] designed self-critical attention (SCAL), which guides model training by evaluating the attention map’s quality and providing strong supervision signals.

However, it is worth noting that these models are convoluted in a limited acceptance domain and cannot effectively capture global context information. Based on these problems, some researchers [29,30] proposed self-attention models. J. Si et al. [7] posed a dual attention matching network based on inter-category and intra-category attention to capture information of human Re-ID video sequences. Dual attention [31] introduced a self-attention mechanism to capture features that depend on the spatial and channel dimension. CBAM [32] is a light and effective network. It can collect the local details information and combine them with global critical information, which can make the image features more representative. 

The first part we want to improve is the attention mechanism module of the model. Based on a lot of research on attention mechanism algorithms, after the position attention module was proposed, we found that the position attention module was also effective. In addition, we observed that the cascaded modules were more effective than the parallel modules according to experiments. Therefore, we connected the three modules of channel attention mechanism, positional attention and spatial attention in series, and obtained the best series order through experiments, that is, CPSA Attention. 

In our opinion, these features of bags and shoes are also within the conv window; however, standard non-attention methods pay more attention to the physical features of pedestrians. This can be proved by the visualization in the Section 6. At first, we found that channel, position and spatial-wise attention (CPSA) had better performance. After comparing the hot map results with other algorithms, we found that CPSA paid more attention to the features of people’s clothes, bags and shoes, while other algorithms paid little attention to these features. Therefore, in our opinion, peoples’ clothes, bags and shoes are inconspicuous local features.

CPS-Attention is a lightweight attention mechanism implemented in tandem. It can extract multi-level features, including the channel, position and spatial features of different depths. Precisely, it can sensitively capture essential details, such as bags and shoes. We found that these three modules were complementary and enabled the network to capture vital information adaptively.

### 2.2. Loss Function for DBA-Net

In addition, there is no doubt that the loss function plays a vital role in the process of model optimization. A good loss function can greatly improve the performance of the network. Loss functions commonly used in Re-ID tasks can be divided into classification loss (ID loss) and metric losses. For the former, Zheng et al. [33] proposed ID differentiated embedding (IDE) to train the model, which is fine-tuned based on the ImageNet [34] pre-training model. Since IDE is trained by classification loss, it is also called ID loss of pedestrian Re-ID. 

However, the performance of models trained only by ID loss is inadequate. Therefore, Chen et al. [14,28,35,36] combined ID loss and triplet loss to optimize the model and achieved good results. However, they only concentrated on positive samples and ignored the samples’ internal structure. Wang et al. [15] considered that internal structure information between samples is helpful to optimize the model better. Gong et al. proposed WRLL [16], which had better adaptability to different datasets.

Furthermore, we notice that the model trained by increasing the network’s output branches properly was robust. Most researchers make improvements based on the global branch of ResNet-50. At the same time, some researchers have proposed a multi-branch strategy. Therefore, we hope to use a multi-branch strategy based on the attention mechanism. We tried different positions and different numbers of branches and design DBA-Net.

Specifically, we used a dual branch strategy to obtain two branches. We made each branch go through GeM [17]. These two branches can optimize the features of different depths in parallel. In this article, we combined ID loss, triplet loss and WRL loss to optimize DBA-Net. We used the same loss function on each branch and found that these three loss functions were complementary. We determined from the fourth part that DBA-Net had excellent robustness.

## 3. Dual Branch Attention Network

At present, some algorithms cannot capture some detailed features, such as knapsacks and shoes. Simultaneously, the recognition of only using the global branch model is inferior to the input pedestrian image. In this case, we designed DBA-Net. We first show the overall structure of the DBA-Net and then analyze the CPSA module. Finally, we introduce dual branch strategy and loss functions.

### 3.1. Architecture of DBA-Net

**Figure 2:**
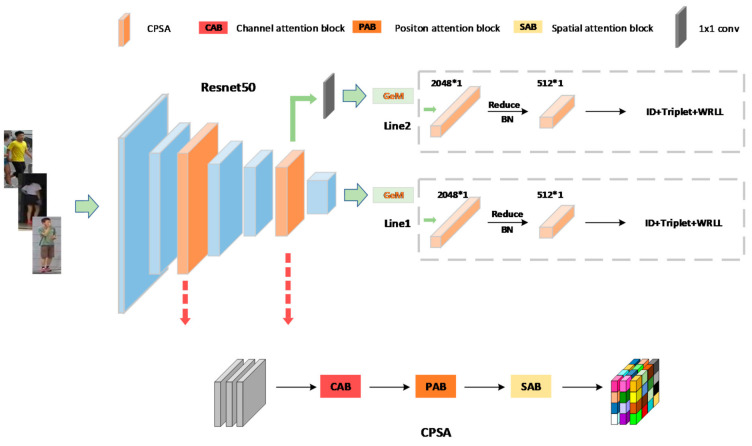
The overall design of DBA-Net. The orange block (left) represents the attention module.

In Re-ID tasks, ResNet-50 [8,13,17,20,37,38,39,40] is the model commonly used as it is simple and effective. For the convenience of comparison with other algorithms, we still use ResNet-50 as the DBA-Net’s backbone network. Figure 2 shows the network’s overall framework. It comprises four parts: ResNet-50, CPS-Attention, GeM and Triplet-WRLL. We embed the CPSA module behind the first layer and the fourth layer.

The CPSA module consists of three small modules (CAB, PAB and SAB). This is a lightweight attention mechanism implemented through tandem. It can effectively capture discriminant features of different levels. We will introduce their specific structure below. To make the model capture detailed features, such as shoes and bags, we not only use the global network branch but introduce another branch after the fourth layer. Each branch is processed by the GeM layer. Based on ID loss, we also use triplet loss and WRLL to optimize the two branches. Specifically, we put Triplet-WRLL behind the GeM layer and use ID loss at the back of the full connection layer.

### 3.2. CPSA Module

Inspired by [6,14], we designed CPSA to extract abundant features and capture multi-level features of the channel, position and spatial. We found that these three modules were complementary. The model can capture the essential details of a pedestrian’s backpack and shoes. In other words, CPSA enhances the representation ability of the model to pedestrian characteristics.

**Figure 3:**
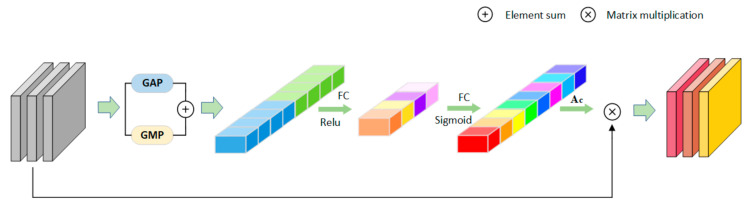
The structure of CAB.

Channel-Wise Attention Block: Figure 3 is the channel attention framework. CAB can effectively perceive the relationship between channels and simulate the interdependence between convolution feature channels. We use average pooling (to determine the range) and maximum pooling (to determine the difference part) in parallel to compress the spatial dimension of feature maps. Then, they produce two different one-dimensional context descriptors: M and N. They are integrated through the attention mechanism [41] to obtain the channel attention map $A_{c}$. Figure 3 presents the architecture of the CAB block. The input channel is compressed during convolution. The calculation formula of channel-wise attention vector is:

(1) $$A=\sigma \left(n_{1}\delta \left(n_{1}M\right)+n_{2}\delta \left(n_{2}N\right)\right)$$

where $n_{1}$ and $n_{2}$ are parameters of the FC layer. *σ* and *δ* represent the Sigmoid function and *ReLU* function, respectively. The obtained $A_{c}$ should be further combined with an original feature map through channel multiplication. CAB can enhance more useful information channels and suppress less useful channels at the same time.

**Figure 4:**
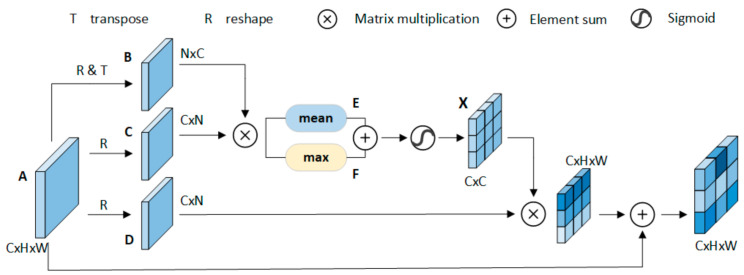
The structure of PAB.

Position-Wise Attention Block: Figure 4 is a position-wise attention framework. PAB can represent the position relationship between each pixel in input feature maps and aggregate semantically related pixels. First, the input feature maps $A_{i},A_{j}\in R^{C\times H\times W}$ are preprocessed to get feature maps $B,C,D\in R^{C\times H\times W}$. Then, we multiply feature maps B, C and calculate the value of the mean (average of each column) and max (maximum value for each column). Finally, we obtained the position-wise attention map $X_{ij}\in R^{C\times C}$ by combining $E_{ij},F_{ij}\in R^{C\times C}$ and the *softmax* function.

(2) $$E_{ij}=\max\left(A_{i}⊗A_{j}\right),F_{ij}=\mathrm{mean}\left(A_{i}⊗A_{j}\right)$$

(3) $$X_{ij}=\frac{\exp\left(E_{ij}+F_{ij}-2\left(A_{i}⊗A_{j}\right)\right)}{\sum_{i=1}^{C}\exp\left(E_{ij}+F_{ij}-2\left(A_{i}⊗A_{j}\right)\right)}$$

where $X_{ij}$ represents the influence of *i*-th channel on the *j*-th channel. We calculate the average value and maximum value of each column in feature maps. Then, we adjust the output size to $R^{C\times C}$. We observed that the fine-grained features will be ignored when using the maximum function alone. Therefore, we also use the average function in parallel.

**Figure 5:**
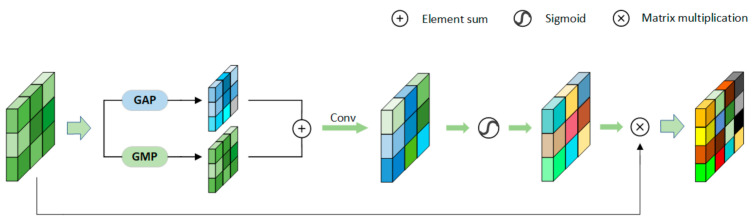
The structure of SAB.

Spatial-Wise Attention Block: Figure 5 is the spatial-wise attention framework. SAB is a supplement to CAB and PAB. It can capture more semantic information in spatial dimension. To obtain feature maps of spatial dimension, we let input feature maps go through two pooling layers (maximum pooling and average pooling) to obtain powerful feature maps of different spaces. Then, the feature maps in different spaces are aggregated to obtain the spatial attention map $Wn\in R^{H\times W}$. SAB can strengthen input features and enhance the consistency of spatial correlation estimation.

### 3.3. Dual Branch with Generalized Mean Pooling 

In Re-ID tasks, we need to solve problems of similar appearance and occlusion. The traditional methods pay less attention to sub-salient information. Therefore, we propose a multi-scale attention mechanism network with dual branches to coordinate the salient and sub-salient information. Specifically, we not only use the global network branch but also introduce another branch after the fourth layer. Each branch is processed by the GeM layer.

The original framework on the basic framework of ResNet-50 only has a branch based on the overall situation. The difference is that we introduce the second branch after the fourth layer. The two branches use the same loss function to train the network in parallel. We use the characteristics of the global branch to test. The double branch strategy can better optimize the model parameters to obtain a more robust deep learning model. Experiments on three public datasets showed that the model trained by this dual branch strategy was robust. We will present the results of DBA-net in the later experiments.

The max-pooling layer and average pooling layer can eliminate redundant information and retain the main features; however, they cannot capture specific areas’ features. Therefore, we use adaptive pooling layer on the two branches, namely generalized mean pooling (GeM) [17]. The following is the formula:

(4) $$f={\left[f_{1}\ldots f_{k}\ldots f_{K}\right]}^{T},f_{k}={\left(\frac{1}{\left|X_{k}\right|}\sum_{x_{i}\in X_{k}}x_{i}{}^{p^{k}}\right)}^{\frac{1}{p^{k}}}$$

where $p^{k}$ is a hyperparameter that can be adjusted when we train the model. When $p^{k}$ →∞, its function is similar to maximum pooling. When $p^{k}$ →1, its function is similar to average pooling.

### 3.4. Loss Function 

In this section, we mainly introduce the adaptive weighted rank list loss (WRLL). Based on ID loss, we also employ triplet loss function. The combined use of them can increase the network’s ability to distinguish similar features.

We present the symbols and their meanings that we use. $X={\left\{\left(x_{i},y_{i}\right)\right\}}_{i=1}^{N}$ is the training set. $\left(x_{i},y_{i}\right)$ are the sample and label with serial number *i*. The samples in the training set totality is *c* and $y_{i}\in \left[1,2,\ldots ,C\right].\;{\left\{\left(x_{i}^{c}\right)\right\}}_{i=1\;}^{N_{c}}$ represents all samples. $P_{c,i\;}^{*}$ represents positive samples. $N_{c,i\;}^{*}$ represent negative samples. Given an image $X_{i}$, our task is to become closer to the positive sample points and to obtain a hypersphere with radius α−m. We need to train all positive sample points together. The following is the calculation formula:

(5) $$L_{m}\left(x_{i},x_{j};f\right)=\left(1-y_{ij}\right)\left|\alpha -d_{ij}\right|+y_{ij}\left|d_{ij}-\left(\alpha -m\right)\right|\;$$

(6) $$L_{p}\left(x_{i}^{c};f\right)=\frac{1}{\left|P_{c,i}^{*}\right|}\underset{x_{j}^{c}\in P_{c,i}^{*}}{\sum }L_{m}\left(x_{i}^{c};x_{j}^{c};f\right)$$

where $d_{ij}=f\left(x_{i}\right)-f{\left(x_{j}\right)}_{2}$ represents the cosine distance between one sample and other samples. To separate the negative sample point from the input as much as possible, we keep a distance of more than *m* between the positive sample point and the negative sample point. We have two hyperparameters (α, m).

The number of positive samples in each batch was less than that of the negative samples. To maintain the generalization performance of network, we used the *softmin* function to assign weights adaptively. The following is the calculation formula:

(7) $$w_{ij}=\frac{\exp\left(-d_{ij}{}^{n}\right)}{\sum_{x_{j}^{c}\in N_{c,i}^{*}}\exp\left(-d_{ij}{}^{n}\right)}$$

where $d_{ij}{}^{n}$ is the Euclidean distance between the input image and the negative samples. This formula represents the process of mining hard samples with adaptive weighting. When there are samples that hard to identify, the adaptive weight will dynamically allocate weight according to the distance between two samples. In Formula (9), we need adjust the parameter τ to achieve the best effect for different datasets. Therefore, we use a more convenient and flexible weighting strategy.

(8) $$RLL_{w_{ij}}=\exp\left(\tau \left(\alpha -d_{ij}\right)\right),\;x_{j}^{k}\epsilon N_{c,i}^{*}$$

where τ is the slope to control the change of weights. To balance the distance between negative samples point and input, the distance between them should be above α.

Similar to the above, the following is the negative sample loss function formula.

(9) $$L_{n}\left(x_{i}^{c};f\right)=\underset{x_{j}^{k}\epsilon |N_{c,i|}^{*}}{\sum }\frac{w_{ij}}{\sum_{x_{j}^{k}\epsilon |N_{c,i}^{*}|}w_{ij}}L_{m}\left(x_{i}^{c};x_{j}^{c};f\right)$$

We optimize both the positive and negative loss functions at the same time.

(10) $$L_{wrll}\left(x_{i}^{c};f\right)=L_{p}\left(x_{i}^{c};f\right)+L_{n}\left(x_{i}^{c};f\right)$$

Finally, we use the triplet loss function simultaneously and obtain the following learning strategy:

(11) $$L_{line1}=L_{line2}=L_{id}+w_{1}L_{wrll}+w_{2}L_{triplet}$$

(12) $$L_{total}=L_{line1}+0.35L_{line2}$$

Among them, $L_{id}$ is a cross-entropy loss function. $L_{triplet}$ is a triplet loss function. $L_{line1}$ and $L_{line2}$ both use the same loss function to optimize. We need to fine-tune the coefficients $w_{1}$ and $w_{2\;}$.

## 4. Experiment

The experimental environment is Pytorch0.4.1, and the server is Tesla V100 GPUs. We evaluated DBA-Net on three large public datasets: Market-1501 [18], DukeMTMC-ReID [19] and Cuhk-03 [2]. First, we compare DBA-Net’s performance with the latest methods. Then, we show the relevant hyper-parameters processing and ablation experiments. Finally, we make a visual analysis of the network.

### 4.1. Dataset Description

Datasets: Through investigation and collection, we decided to use three authoritative datasets for our research, namely the three mentioned above. We have a detailed introduction in Table 1. Specifically, Market1501 contains 32,668 images of 1501 labeled persons of six camera views; 12,936 images of 751 identities were randomly selected as the training set, and 19,732 images from 750 identities were used as the testing set. As a large-scale dataset, DukeMTMC-ReID has 36,411 images of 1404 identities from eight countries; 16,522 images of 702 identities were selected as the training set, and 19,889 images from 702 identities were used as the testing set. Cuhk-03 is a small-scale re-identification dataset with 14,088 images of 1467 identities.

Evaluation Metrics: We used mAP and the cumulative matching feature (CMC) to evaluate the performance of DBA-Net. These are widely recognized in Re-ID task, for the CMC curve. The abscissa of this curve is Rank-*n* (*n* = 1, 3, 5···), in which the ordinate is the corresponding precision. We can intuitively find the recognition accuracy of Rank-n through the CMC curve. Rank-n is the possibility that the target is correctly retrieved in the top n recognition. mAP intuitively presents the average accuracy of correct retrieval during testing. It can reflect the overall performance of models.

Other details: We used horizontal flipping, random cropping [42] and random erasing [43] to preprocess the image. The size of the input images was adjusted to 256 × 128. Our backbone network was ResNe-50 pre-trained on ImageNet [34]. The baseline network was BagTricks [10,20] that only used the cross-entropy loss function. We used the Adam algorithm optimizer to train models. The initial learning rate (Lr) and weight decay were both 0.0005. Table 2 presents the setting of the learning rate. *t* represents the number of epochs.

### 4.2. Hyper-Parameter Experimental Analysis

In this section, we analyze the parameters of loss functions. Since there are four parameters, we adopted the following strategy. First, we only used the WRL loss function and determined the appropriate values of α and m through comparative experiments. Then, we added a triplet loss function and analyzed $w_{1}$ and $w_{2}$. Finally, we used Triplet loss and WRL loss together to analyze α and m again.

**Figure 6:**
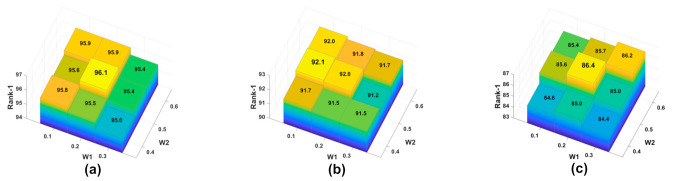
Loss function optimization for DBA-Net on three datasets. (**a**) The Market-1501 dataset. (**b**) The DukeMTMC-ReID dataset. (**c**) The Cuhk-03 dataset.

Coefficient $w_{1}$ and $w_{2}$: With the loss functions, we found that the experimental results of different coefficient combinations were different. We needed to adjust the loss functions’ coefficients properly. Following the parameter adjustment method of GLWR [16], we used this method of controlling variables. The initial values were $w_{1}$ = 0.5, $w_{2}$ = 0.5. For example, we fixed the parameter $w_{2}$ = 0.5. We changed the value of $w_{2}$ and selected the best experimental result through extensive experiments. Every time we changed by 0.1 to find the approximate value of $w_{2}$. After that, we changed by 0.05 each time to obtain the final value of $w_{2}$. Then, we fixed the parameter $w_{1}$ and found the optimal value of $w_{2}$. Figure 6 shows the optimized results.

We can intuitively find that the coefficients $w_{1}$ and $w_{2}$ had a significant influence on the model from Figure 6. Through extensive experiments, we found our model had the best performance when $w_{1}$ = 0.2, $w_{2}$ = 0.5.

**Figure 7:**
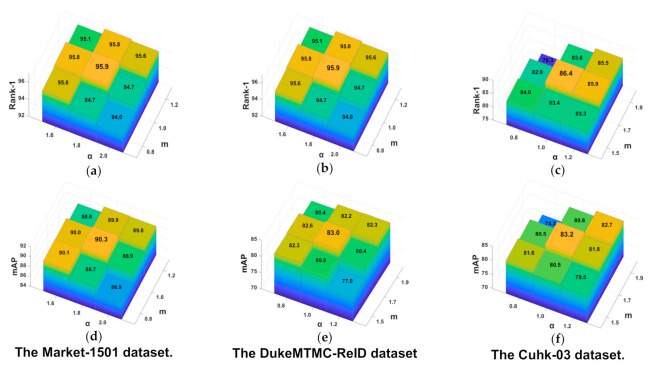
Hyper-parameter optimization for DBA-Net on three datasets. (**a**) The Market-1501 dataset. (**b**) The DukeMTMC-ReID dataset. (**c**) The Cuhk-03 dataset. (**d**) The Market-1501 dataset. (**e**) The DukeMTMC-ReID dataset. (**f**) The Cuhk-03 dataset.

Hyper-parameters m and α: Furthermore, the parameters m and α in Equation (5) also had a significant influence on the performance of DBA-Net. The size of α−m determined by the dataset represent the radius of the hypersphere. We need to adjust it properly to ensure good performance of the model. Therefore, we performed many comparative experiments on these two parameters. We also used the control variable method. Figure 7 presents the optimized results.

As shown in Figure 7, the parameters α and m had a great influence on DBA-Net. According to a large number of experimental results, we have the following findings: For market-1501, DBA-Net had the performance best when α = 1.8, and m = 1.0. For DukeMTMC-ReID, DBA-Net had the performance best when α = 2.0, and m = 1.2. For CUHK-03, DBA-Net had the performance best when α = 1.7, and m = 1.0.

### 4.3. Comparison with State-of-the-Art Methods

We compared DBA-Net with the state-of-the-art (SOTA) approaches: AGW [1], CAM [4], ABD-Net [6], BagTricks [9], AANet [10], RAG-SC [12], SGSC [14], GRLL [16], MGN [20], GD-Net [21], IANet [23], Pyramid [24], SONA [27], SCAL [28], Auto-ReID+ [44] and Ms-Mb [45]. We did not adopt the re-ranking trick for the sake of fairness. The ResNet-50 [42] is the backbone. Pyramid [24] is an exception, where the backbone network is ResNet-101.

Results on Market-1501: We show these results in Table 3. In these methods, it should be pointed out that Pyramid [24] uses a more powerful backbone and complex fringe features. It is a representative of global feature algorithms. SGCS [16] uses a cascading strategy to extract different potential features in each stage, and each stage integrates these features for the final representation. However, the global features ignore the local details. Cascade strategy also greatly increases the complexity of the network. Finally, our approach was more competitive than other SOTA approaches.

Results on DukeMTMC-ReID: We show these results in Table 4. On this dataset, DBA-Net also achieved the best effect on Rank-1. It was 4% higher than SGCS [16]. In addition, our results on lightweight networks were higher than Pyramid [24] by 4%.

Results on CUHK-03: We show these results in Table 5. Compared with the two datasets above, the CUHK-03 dataset was more challenging, as this dataset has few samples and the occlusion problem is serious. However, DBA-Net can capture more detailed features, such as shoes and bags and can accurately retrieve the target pedestrian. DBA-Net surpassed Pyramid [24] by 7.5% in Rank-1 and 8.4% in mAP. In addition, DBA-Net outperformed SGCS [16] and achieved a new SOTA.

We compared DBA-Net against the algorithm with the best current experimental results. Specifically, Ms-Mb [45] achieved 95.8% top-1 accuracy and 88.9% mAP on Market-1501. SGSN [16] achieved 91.0% top-1 accuracy on DukeMTMC-Re-ID. Additionally, SGSN [16] obtained 84.7% top-1 accuracy and 81.0% mAP on CUHK-03, as shown in Table 3, Table 4 and Table 5. Nevertheless, the experimental results of DBA-Net clearly exceeded these. Especially on the CUHK03 dataset, DBA-Net surpassed SGSN [16] by 1.7% in Rank-1 and 2.2% in mAP.

Through comprehensive analysis of these experimental results, DBA-Net had the best performance. DBA-Net’s mAP values on three datasets were 90.3%, 83.1% and 83.2%, which are higher than the above suboptimal network. The model achieved the new SOTA on these three popular public datasets.

### 4.4. Ablation Experiment

In this section, we demonstrate the effectiveness of CPSA, Triplet-WRLL and DBA-Net by ablation experiments. The network performance will decline when the parameter τ (in the part of the loss function) is a large number. We set it to 0.3. Table 6 presents the results of the ablation experiments.

First, we used the baseline of BagTricks [10] that only uses the cross-entropy loss function and kept this as our baseline. Then, we stacked GeM, Triplet, WRLL and CPSA modules on the baseline in turn. We found that each module effectively improved the performance of the network. As we expected, the network trained by the combination of these three loss functions was robust. In particular, WRL loss had outstanding performance in the cuhk-03 (detected) dataset. In addition, CPS-Attention made the network more recognizable to input images. Finally, DBA-Net had the best performance.

**Figure 8:**
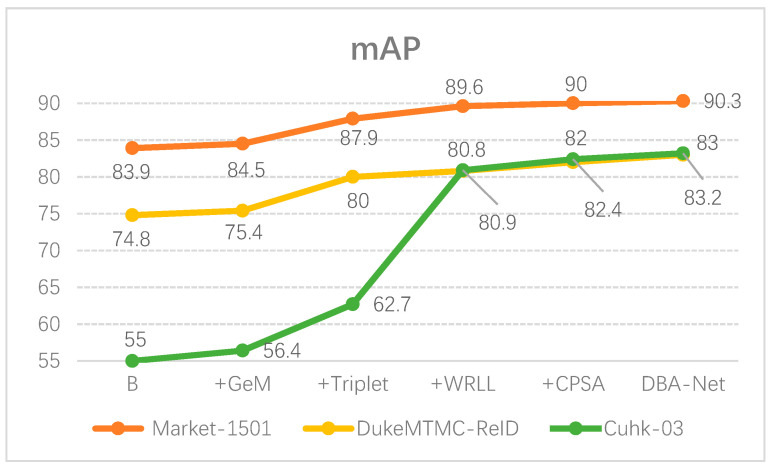
The mAP value of DBA-Net on three datasets.

**Figure 9:**
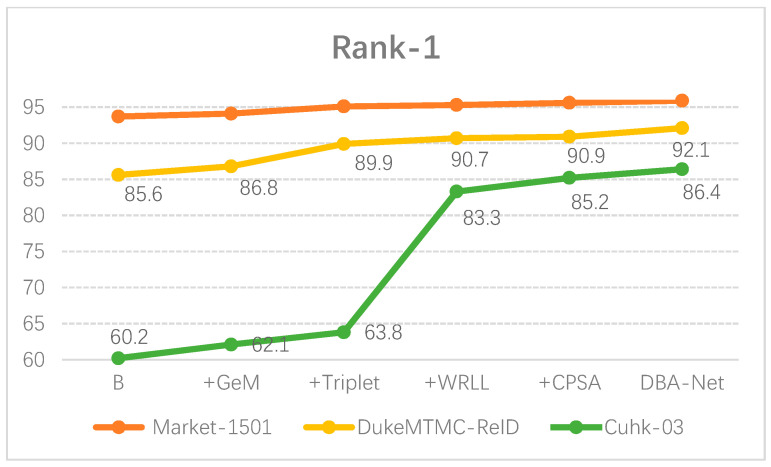
The Rank-1 of DBA-Net on three datasets.

Furthermore, we also made graphs to demonstrate the ablation experiments more intuitively as shown in Figure 8 and Figure 9.

### 4.5. CPS-Attention 

**Figure 10:**
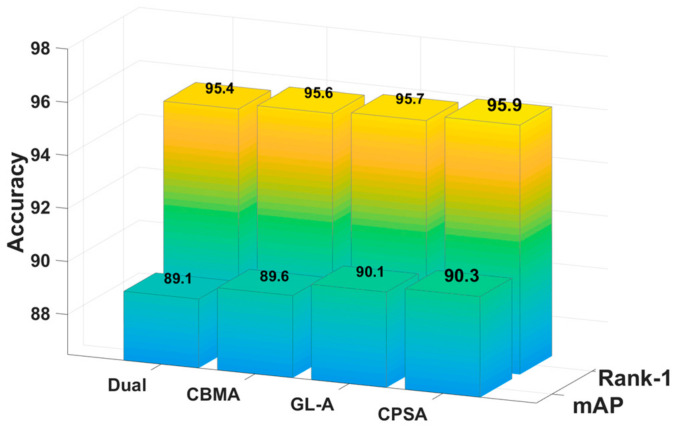
The comparative experiments of CPS-Attention on Market-1501.

**Figure 11:**
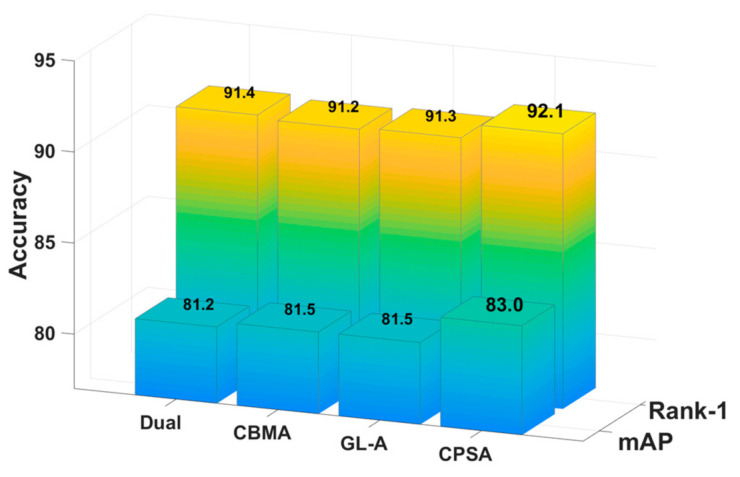
The comparative experiments of CPS-Attention on DukeMTMC-ReID.

**Figure 12:**
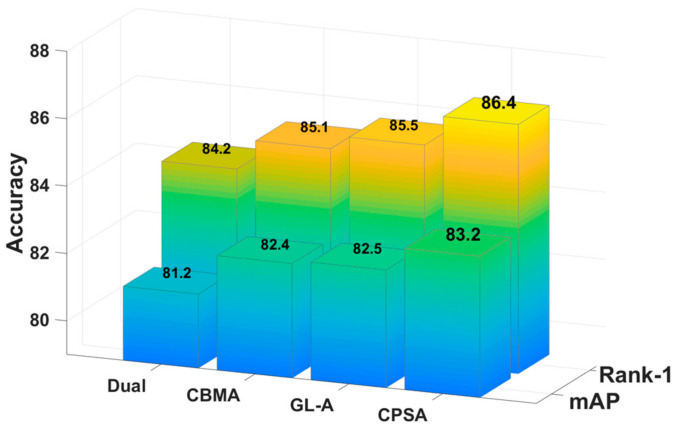
The comparative experiments of CPS-Attention on CUHK-03(detected).

In this section, we prove the effectiveness of CPS-Attention according to comparative experiments. Specifically, we added other advanced attention modules to the same benchmark network for comparison, including Dual attention [31], CBAM [32] and GL-attention [16]. To be fair, these experiments were all conducted on DBA-Net. (Figure 10, Figure 11 and Figure 12) show the comparative experimental results of the attention models.

CPS-Attention combines three modules and focuses on the local features, such as pedestrian’s backpack and shoes. In other words, CPSA enhances the representation ability of the model. For market-1501, CPS-Attention was higher than other attention blocks on mAP and Rank-1. Especially for DukeMTMC-ReID and CUHK-03, CPS-Attention had clearly superior performance. Finally, CPSA demonstrated remarkable improvement on DBA-Net.

## 5. Visualization and Analysis

### 5.1. Loss and CMC Curves 

**Figure 13:**
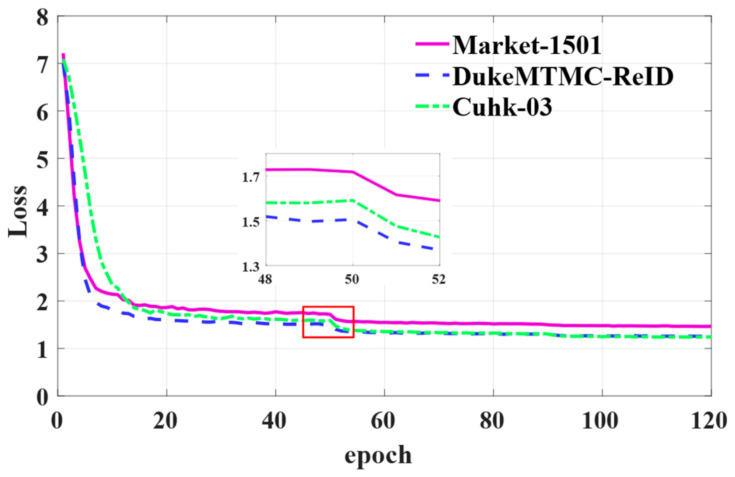
The loss curves of DBA-Net on three datasets.

Figure 13 shows curves of the loss function value in the process of training. These three curves present a steady downward trend, which indicates that the loss value was steadily decreasing. In other words, we were training a stable network. When the epoch is equal to 50, the curves have an apparent downward trend as a result of the change of the learning rate. The specific settings of the learning rate were described in detail above.

**Figure 14:**
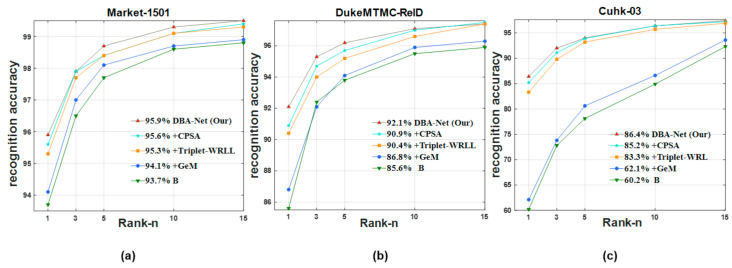
CMC curves of DBA-Net on three datasets. (**a**) The Market-1501 dataset. (**b**) The DukeMTMC-ReID dataset. (**c**) The Cuhk-03 dataset.

We present the CMC curve of DBA-Net on three datasets in Figure 14. GeM, CPSA and Triplet-WRLL improved the performance of the model respectively. Finally, DBA-Net had the best performance. The curves from the bottom to the top correspond to the following experiments: B, B+Gem, B+ Gem+Triplet-WRLL, B+Gem+Triplet-WRLL+CPSA and DBA-Net. The CMC curve directly reflects the overall performance of the model. Figure 14 presents that DBA-Net had excellent robustness.

### 5.2. Visualization 

**Figure 15:**
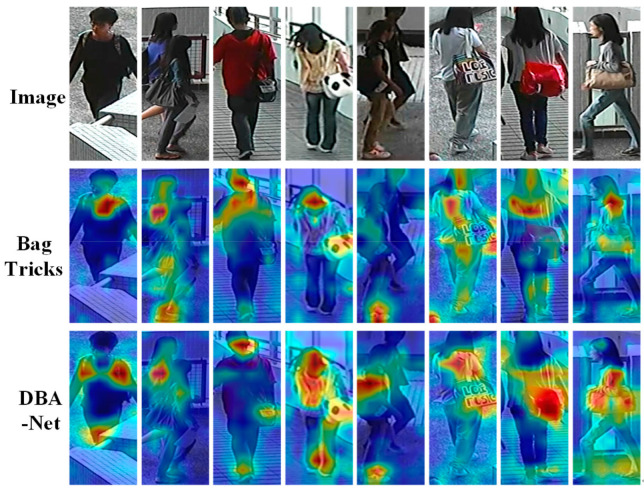
Heat maps of BagTricks and DBA-Net. Images in the same column share the same identity.

We used Grad-CAM [46] as the tool to assess DBA-Net and BagTricks [10]. According to the output vector, the gradient corresponding to each pixel on each feature map was obtained, which was the gradient map corresponding to the feature map. Finally, this is presented in the form of hot maps. It enables us to find the area most sensitive to the network in the input picture. The deeper red represents more crucial. In Figure 15, we visualize the results of the two models. We show eight representative feature maps. 

In the second pedestrian picture, BagTricks [10] mistakenly focuses on other people’s physical characteristics. In the sixth pedestrian picture, BagTricks pays attention to useless background information. By comparison, DBA-Net can accurately lock the critical part of the body features. In the third and seventh pedestrian pictures, DBA-Net captures the obvious features of bags. In the third to fifth pedestrian pictures, DBA-Net captures the local features of pedestrians’ bags and shoes. In a few words, DBA-Net pays more attention features of people clothes, bags and shoes. It pays less attention to irrelevant background information and more attention to essential features than BagTricks [10].

**Figure 16:**
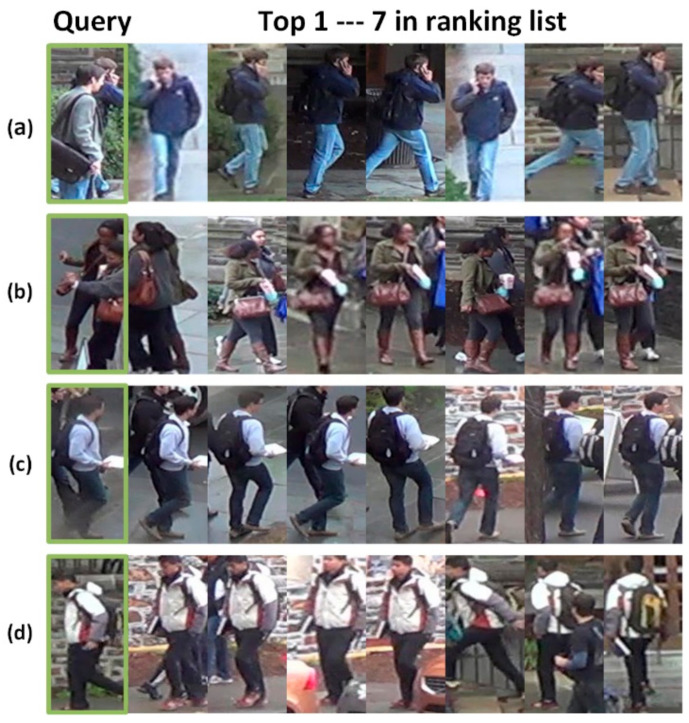
Four query images and query results of DBA-Net on DukeMTMC-ReID. (**a**) Sample 1. (**b**) Sample 2. (**c**) Sample 3. (**d**) Sample 4.

As the problems of occlusion and similar appearance. Figure 1 presents the visualization experiments on BagTricks [10]. There are many mistakes in the process of retrieving pedestrians. Figure 16 presents the visualization experiments on DBA-Net. The pedestrian in the first image is seriously occlude, and the pedestrian in the second and third images is similar to the other pedestrian. However, our model can still be retrieved accurately. The pedestrian in the fourth image is disturbed by background information. DBA-Net can still accurately retrieve the corresponding images for pedestrians. In other words, DBA-Net can effectively alleviate the problems of occlusion, similar appearance and background interference.

## 6. Conclusions

In this paper, we proposed a novel attention network (DBA-Net). We embedded a powerful attention module (CPSA) into the network. Furthermore, we used complementary loss functions (Triplet-WRLL) on the two branches of the network. Finally, extensive experiments proved that DBA-Net achieved advanced performance. In addition, we discovered that the research on Re-ID in the current domain has reached a bottleneck, and it is, thus, difficult to make a breakthrough in evaluation metrics. At present, some researchers are conducting cross domain work and have achieved certain results. However, there is still a huge research space in cross-domain tasks. The research of cross-domain Re-ID is a new research trend. In the future, we intend to apply DBA-Net to the cross-domain Re-ID task.

## Figures and Tables

**Table 1:** Introduction to dataset.

Datasets	Train ID	Images	Test ID	Images	Sum	Cameras
Market-1501	751	12,936	750	19,732	32,668	6
DukeMTMC-ReID	702	16,522	702	19,889	36,411	8
Cuhk-03	767	7356	700	6732	14,088	2

**Table 2:** The setting of the learning rate.

Epoch	Lr	Epoch	Lr
1–10	5.0 × $10^{-4}$ × 0.1 *t*		
11–50	5.0 × $10^{-4}$	161–210	4.0 × $10^{-6}$
51–90	1.0 × $10^{-4}$	211–250	8.0 × $10^{-7}$
91–160	2.0 × $10^{-5}$	251–300	1.6 × $10^{-7}$

**Table 3:** Comparison with the SOTA methods on Market-1501. The best performance is shown in red, and the second best performance is shown in blue.

Methods	Rank-1	mAP
AANet [9] (CVPR2019)	93.9	83.4
IANet [23] (CVPR2019)	94.4	83.1
SONA [27] (CVPR2019)	95.6	88.8
AGW [1] (arXiv2020)	95.1	87.8
ABD-Net [6] (CVPR2019)	95.6	88.3
MGN [20] (ACM2018)	95.7	86.9
CAM [4] (CVPR2019)	94.7	84.5
GD-Net [21] (CVPR2019)	94.8	86.0
BagTricks [10] (CVPR2019)	94.5	85.9
GLWR [16] (IEEE Access 2020)	95.5	88.5
Pyramid [24] (CVPR2019)	95.7	88.2
Auto-ReID+ [44] (Neurocomputing2021)	95.8	88.2
SGSN(4 stages) [16] (CVPR2020)	95.7	88.5
Ms-Mb [45] (Neurocomputing2020)	95.8	88.9
DBA-Net	95.9	90.3

**Table 4:** Comparison with the SOTA methods on the DukeMTMC-ReID. The best performance is shown in red, and the second best performance is shown in blue.

Methods	Rank-1	mAP
AANet [9] (CVPR2019)	87.7	74.3
IANet [23] (CVPR2019)	83.1	73.4
SONA [27] (CVPR2019)	89.5	78.3
AGW [1] (arXiv2020)	89.0	79.6
ABD-Net [6] (CVPR2019)	89.0	78.6
MGN [20] (ACM2018)	88.7	78.4
CAM [4] (CVPR2019)	85.8	72.9
GD-Net [21] (CVPR2019)	86.6	74.8
BagTricks [10] (CVPR2019)	86.4	76.4
SCAL (spatial) [28] (ICCV2019)	89.0	79.6
SCAL (channel) [28] (ICCV2019)	88.9	79.1
GLWR [16] (IEEE Access 2020)	90.7	81.4
Pyramid [24] (CVPR2019)	89.0	79.0
Auto-ReID+ [44] (Neurocomputing2021)	90.1	80.1
SGSN(4 stages) [16] (CVPR2020)	91.0	79.0
Ms-Mb [45] (Neurocomputing2020)	90.8	82.2
DBA-Net	92.1	83.0

**Table 5:** Comparison with the SOTA methods on the CUHK-03(detected). The best performance is shown in red, and the second best performance is shown in blue.

Methods	Rank-1	mAP
SONA [27] (CVPR2019)	79.9	77.3
AGW [1] (arXiv2020)	63.6	62.0
RAG-SC [12] (CVPR2020))	79.6	74.5
MGN [20] (ACM2018)	68.0	66.0
CAM [4] (CVPR2019)	66.6	64.2
BagTricks [10] (CVPR2019)	58.8	56.6
SCAL (channel) [28] (ICCV2019)	71.1	68.6
GLWR [16] (IEEE Access 2020)	82.3	78.9
Pyramid [24] (CVPR2019)	78.9	74.8
Auto-ReID+ [45] (Neurocomputing2021)	78.1	74.2
Ms-Mb [44] (Neurocomputing2020)	75.4	72.9
SGSN(4 stages) [16] (CVPR2020)	84.7	81.0
DBA-Net	86.4	83.2

**Table 6:** Ablation experiments of DBA-Net.

	Market-1501		DukeMTMC-ReID		Cuhk-03	
	Rank-1	mAP	Rank-1	mAP	Rank-1	mAP
B	93.7	83.9	85.6	74.8	60.2	55.0
+GeM	94.1	84.5	86.8	75.4	62.1	56.4
+Triplet	95.1	87.9	89.9	80.0	63.8	62.7
+WRLL	95.3	89.6	90.7	80.8	83.3	80.9
+CPSA	95.6	90.0	90.9	82.0	85.2	82.4
DBA-Net	95.9	90.3	92.1	83.0	86.4	83.2

## Data Availability

We evaluate our network on three large public datasets: Market-1501, DukeMTMC-ReID and Cuhk-03. https://github.com/NEU-Gou/awesome-reid-dataset accessed on 27 August 2021.
